# Dicaesium hexa­mercury hepta­sulfide

**DOI:** 10.1107/S1600536808023477

**Published:** 2008-08-09

**Authors:** Daniel E. Bugaris, James A. Ibers

**Affiliations:** a Department of Chemistry, Northwestern University, 2145 Sheridan Road, Evanston, IL 60208-3113, USA

## Abstract

The title compound, Cs_2_Hg_6_S_7_, crystallizes in a new structure type that is closely related to that of K_2_Zn_6_O_7_. The structure comprises a three-dimensional mercury sulfide network that is composed of channels. These channels, which are along [001], are of two different diameters. The crystal structure contains one Cs, two Hg, and three S atoms in the asymmetric unit. The Cs, one Hg, and one S atom are at sites of symmetry *m*, whereas a second S atom is at a site of symmetry 2*mm*. The Hg atoms are bound to the S atoms in both three- and four-coordinate geometries. The caesium cations occupy the central spaces of the larger diameter channels and exhibit a coordination number of 7.

## Related literature

For literature on related alkali-metal mercury sulfides, see: Sommer & Hoppe (1978 ▶) for *A*_6_HgS_4_ (*A* = K, Rb); Klepp & Prager (1992 ▶) for *A*_2_HgS_2_ (*A* = Na, K); Klepp (1992 ▶) for Na_2_Hg_3_S_4_; Kanatzidis & Park (1990 ▶) for K_2_Hg_3_S_4_ and K_2_Hg_6_S_7_. For literature on other compounds with Hg—S or Cs—S bonds, see: Gulay *et al.* (2002 ▶); Rad & Hoppe (1981 ▶); Iwasaki (1973 ▶); Kinoshita *et al.* (1985 ▶); Bronger & Hendriks (1980 ▶). The isoformular, but not isotypic, compound K_2_Zn_6_O_7_ was reported by Wambach & Hoppe (1978 ▶). For synthetic details, see: Sunshine *et al.* (1987 ▶). For computational details, see: Brown & Altermatt (1985 ▶); Gelato & Parthé (1987 ▶); Le Page (1988 ▶); Brese & O’Keeffe (1991 ▶); Spek (2003 ▶).

## Experimental

### 

#### Crystal data

Cs_2_Hg_6_S_7_
*M* *_r_* = 1693.78
Tetragonal, 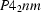

*a* = 14.063 (3) Å
*c* = 4.1895 (18) Å
*V* = 828.6 (4) Å^3^
*Z* = 2
Mo *K*α radiation
μ = 60.56 mm^−1^
*T* = 215 (2) K
0.44 × 0.06 × 0.04 mm

#### Data collection

Bruker SMART 1000 CCD diffractometer
Absorption correction: face-indexed, numerical (*SADABS*; Sheldrick, 2006 ▶) *T*_min_ = 0.011, *T*_max_ = 0.131
8644 measured reflections
956 independent reflections
907 reflections with *I* > 2σ(*I*)
*R*_int_ = 0.052

#### Refinement

*R*[*F* ^2^ > 2σ(*F* ^2^)] = 0.034
*wR*(*F* ^2^) = 0.082
*S* = 1.23
956 reflections
43 parameters
1 restraint
.
Δρ_max_ = 2.17 e Å^−3^
Δρ_min_ = −2.29 e Å^−3^
Absolute structure: Flack (1983 ▶), 389 Friedel pairs
Flack parameter: 0.00 (3)

### 

Data collection: *SMART* (Bruker, 2003 ▶); cell refinement: *APEX2* (Bruker, 2006 ▶); data reduction: *SAINT* (Bruker, 2006 ▶); program(s) used to solve structure: *SHELXS97* (Sheldrick, 2008 ▶); program(s) used to refine structure: *SHELXL97* (Sheldrick, 2008 ▶); molecular graphics: *CrystalMaker* (Palmer, 2008 ▶); software used to prepare material for publication: *SHELXL97*.

## Supplementary Material

Crystal structure: contains datablocks I, global. DOI: 10.1107/S1600536808023477/wm2184sup1.cif

Structure factors: contains datablocks I. DOI: 10.1107/S1600536808023477/wm2184Isup2.hkl

Additional supplementary materials:  crystallographic information; 3D view; checkCIF report

## Figures and Tables

**Table:** 

Hg1—S1	2.488 (4)
Hg1—S1^i^	2.540 (5)
Hg1—S1^ii^	2.549 (5)
Hg1—S3^iii^	2.7754 (15)
Hg2—S3	2.367 (7)
Hg2—S2	2.377 (5)
Hg2—S3^iv^	3.004 (7)
Cs1—S1^v^	3.526 (5)
Cs1—S1^ii^	3.526 (5)
Cs1—S1^vi^	3.561 (5)
Cs1—S1^i^	3.561 (5)
Cs1—S2^vii^	3.566 (4)
Cs1—S3^iv^	3.609 (7)
Cs1—S3	3.627 (7)

**Table:** 

S1—Hg1—S1^i^	121.0 (2)
S1—Hg1—S1^ii^	120.6 (2)
S1^i^—Hg1—S1^ii^	110.83 (15)
S1—Hg1—S3^iii^	104.78 (15)
S1^i^—Hg1—S3^iii^	96.43 (18)
S1^ii^—Hg1—S3^iii^	95.74 (17)
S3—Hg2—S2	168.5 (3)
S3—Hg2—S3^iv^	101.9 (2)
S2—Hg2—S3^iv^	89.7 (2)

Symmetry codes: (i) 
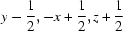
; (ii) 
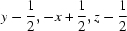
; (iii) 
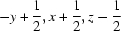
; (iv) 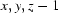; (v) 
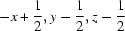
; (vi) 
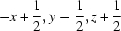
; (vii) 
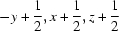
.

**Fig. 1.**
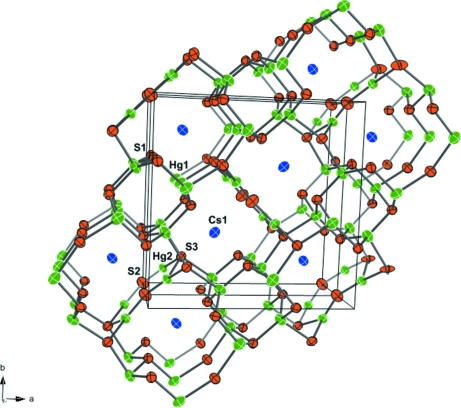
A view down [001] of the unit cell of Cs2Hg6S7, with displacement ellipsoids at the 95% probability level.

## supplementary crystallographic information

### Comment

Four structure types are found in alkali metal-mercury-sulfur ternary systems: *A*_6_HgS_4_ (*A* = K, Rb) exhibit discrete HgS_4_ tetrahedra with intercalated alkali-metal cations (Sommer & Hoppe, 1978). *A*_2_HgS_2_ (*A* = Na, K) possess nearly linear HgS_2_ units with alkali-metal cations occupying the voids (Klepp & Prager, 1992). The compounds Na_2_Hg_3_S_4_ (Klepp, 1992) and K_2_Hg_3_S_4_ (Kanatzidis & Park, 1990) feature infinite [Hg_3_S_4_]^2-^ units parallel to (100) separated by alkali-metal cations. K_2_Hg_6_S_7_ has a three-dimensional mercury sulfide network composed of two types of channels, with the potassium cations occupying the spaces in the larger-diameter channels (Kanatzidis & Park, 1990).

Fig. 1 shows the unit cell of Cs_2_Hg_6_S_7_. Similar to K_2_Hg_6_S_7_, there is a three-dimensional mercury sulfide network containing two types of channels along [001]. There are two types of coordination geometries for the mercury atoms: Hg1 is four-coordinate with a distorted tetrahedral geometry and Hg2 is three-coordinate with a T-shaped planar geometry. The Hg1—S bond distances of 2.488 (4)-2.775 (2) Å are comparable to those of 2.345 (8)-2.718 (4) Å in K_2_Hg_6_S_7_. The Hg2—S bond distances are 2.367 (7), 2.377 (5), and 3.004 (7) Å. In K_2_Hg_6_S_7_, the Hg2 atom is considered to have a two-coordinate, nearly linear geometry. The Hg2 atom in Cs_2_Hg_6_S_7_ can also be viewed as having an approximately linear bonding environment if the Hg2—S coordination distance of 3.004 (7) Å is considered to be non-bonding. However, there are some examples in the literature of longer than expected Hg—S bonds in extended solid-state structures, including 2.92 (1) Å in Hg_4_SiS_6_ (Gulay *et al.*, 2002), and 2.98 (1) Å in BaHgS_2_ (Rad & Hoppe, 1981). Long Hg—S bond distances of greater than 2.9 Å are more common in coordination compounds, including 2.965 (4) Å in Hg(S_2_CNEt_2_)_2_ (Iwasaki, 1973), 3.137 (6) Å in Hg_2_(S_2_CNEt_2_)_4_ (Iwasaki, 1973), and 2.991 (3) Å and 3.322 (4) Å in [CoHg(SCN)_4_{P(C_6_H_5_)_3_}_2_]_2_ (Kinoshita *et al.*, 1985). Therefore, we consider the Hg2 atom in Cs_2_Hg_6_S_7_ to be three-coordinate with a T-shaped planar geometry. The Cs1 atoms, found in the centers of the larger-diameter channels, are seven-coordinate, with Cs—S distances of 3.526 (5)-3.627 (7) Å, as compared to 3.595 (7)-3.701 (6) Å in Cs_2_Zn_3_S_4_ (Bronger & Hendriks, 1980).

Because there is no S—S bonding in Cs_2_Hg_6_S_7_, a formal oxidation state of -II can be assigned to the S atoms. Bond valence sums (Brown & Altermatt, 1985; Brese & O'Keeffe, 1991) of Hg1 = 1.95, Hg2 = 1.84, and Cs1 = 1.13 confirm the formal oxidation states of +II for both Hg atoms, and +I for Cs.

Although they possess structural similarities, Cs_2_Hg_6_S_7_ and K_2_Hg_6_S_7_ are not isostructural. Cs_2_Hg_6_S_7_ belongs to space group *P*4_2_*nm* and K_2_Hg_6_S_7_ belongs to space group *P*42_1_*m*. Another related compound is K_2_Zn_6_O_7_, which crystallizes in space group *P*4_2_*nm* (Wambach & Hoppe, 1978). However, the *ADDSYM* algorithm (Le Page, 1988) in *PLATON* (Spek, 2003) suggests that K_2_Zn_6_O_7_ properly belongs to space group *P*4_2_/*mnm*. Apparently, Cs_2_Hg_6_S_7_ is not isostructural to K_2_Zn_6_O_7_ and instead crystallizes in a new structure type.

### Experimental

Cs_2_Hg_6_S_7_ was initially obtained in an attempt to produce a Cs/Hg/U/S quaternary phase. Cs_2_Hg_6_S_7_ was then synthesized rationally from a solid-state reaction of Cs_2_S_3_ (0.13 mmol), HgS (Alfa Aesar, 0.21 mmol), and S (Mallinckrodt, 99.6%, 0.44 mmol). CsI (Aldrich, 99.9%, 0.34 mmol) was added to aid in the crystallization of the final product. The Cs_2_S_3_ reactive flux (Sunshine *et al.*, 1987) was prepared by the stoichiometric reaction of Cs (Strem Chemicals, 99.5%) and S in liquid NH_3_ at 194 K. The reactants were loaded into a fused-silica tube under an Ar atmosphere in a glove box. The tube was evacuated to 10 ^-4^ Torr, sealed, and then placed in a computer-controlled furnace. The sample was heated to 1123 K in 24 hours, kept at 1123 K for 120 h, cooled at 5 K h^-1^ to 473 K, and then cooled to 293 K in 2 hours. The resulting black needles were washed with *N,N*-dimethylformamide. The yield was about 80% based on Hg.

### Refinement

The structure was standardized by means of the program *STRUCTURE TIDY* (Gelato & Parthé, 1987). The highest peak is 1.60 Å from atom Hg1 and the deepest hole is 1.03 Å from atom S3.

### Crystal data

**Table:**

Cs_2_Hg_6_S_7_	*Z* = 2
*M**_r_* = 1693.78	*F*_000_ = 1404
Tetragonal, *P*4_2_*n**m*	*D*_x_ = 6.789 Mg m^−^^3^
Hall symbol: P 4n -2n	Mo *K*α radiation λ = 0.71073 Å
*a* = 14.063 (3) Å	Cell parameters from 5893 reflections
*b* = 14.063 (3) Å	θ = 2.9–28.1º
*c* = 4.1895 (18) Å	µ = 60.56 mm^−^^1^
α = 90º	*T* = 215 (2) K
β = 90º	Needle, black
γ = 90º	0.44 × 0.06 × 0.04 mm
*V* = 828.6 (4) Å^3^	

### Data collection

**Table:**

Bruker SMART 1000 CCD diffractometer	956 independent reflections
Radiation source: fine-focus sealed tube	907 reflections with *I* > 2σ(*I*)
Monochromator: graphite	*R*_int_ = 0.052
*T* = 215(2) K	θ_max_ = 28.1º
ω scans	θ_min_ = 2.1º
Absorption correction: numerical(SADABS; Sheldrick, 2006)	*h* = −17→17
*T*_min_ = 0.011, *T*_max_ = 0.131	*k* = −17→17
8644 measured reflections	*l* = −5→5

### Refinement

**Table:**

Refinement on *F*^2^	Secondary atom site location: difference Fourier map
Least-squares matrix: full	*w* = 1/[σ^2^(*F*_o_^2^) + (0.0235*P*)^2^ + 40.9819*P*] where *P* = (*F*_o_^2^ + 2*F*_c_^2^)/3
*R*[*F*^2^ > 2σ(*F*^2^)] = 0.034	(Δ/σ)_max_ < 0.001
*wR*(*F*^2^) = 0.082	Δρ_max_ = 2.17 e Å^−^^3^
*S* = 1.23	Δρ_min_ = −2.29 e Å^−^^3^
956 reflections	Extinction correction: *SHELXL97* (Sheldrick, 2008), Fc^*^=kFc[1+0.001xFc^2^λ^3^/sin(2θ)]^-1/4^
43 parameters	Extinction coefficient: 0.00320 (17)
1 restraint	Absolute structure: Flack (1983), 389 Friedel pairs
Primary atom site location: structure-invariant direct methods	Flack parameter: 0.00 (3)

### Special details

**Table:**

Experimental. The absorption correction program used the face indices and crystal dimensions that were supplied.

### Fractional atomic coordinates and isotropic or equivalent isotropic displacement parameters (Å^2^)

**Table:**

	*x*	*y*	*z*	*U*_iso_*/*U*_eq_	
Hg1	0.15251 (5)	0.57410 (5)	0.22339 (19)	0.0216 (2)	
Hg2	0.09922 (5)	0.09922 (5)	0.3162 (3)	0.0189 (3)	
Cs1	0.33108 (8)	0.33108 (8)	0.2149 (5)	0.0197 (3)	
S1	0.0207 (3)	0.6920 (3)	0.2220 (13)	0.0151 (7)	
S2	0.0000	0.0000	0.000 (2)	0.0193 (19)	
S3	0.1828 (3)	0.1828 (3)	0.7187 (18)	0.0153 (11)	

### Atomic displacement parameters (Å^2^)

**Table:**

	*U*^11^	*U*^22^	*U*^33^	*U*^12^	*U*^13^	*U*^23^
Hg1	0.0248 (4)	0.0215 (4)	0.0184 (4)	0.0052 (3)	0.0002 (3)	−0.0002 (3)
Hg2	0.0180 (3)	0.0180 (3)	0.0206 (5)	−0.0015 (4)	−0.0032 (3)	−0.0032 (3)
Cs1	0.0216 (5)	0.0216 (5)	0.0157 (7)	0.0037 (5)	−0.0013 (6)	−0.0013 (6)
S1	0.0134 (18)	0.0160 (18)	0.0158 (18)	−0.0006 (14)	−0.0002 (19)	−0.0017 (19)
S2	0.024 (3)	0.024 (3)	0.011 (4)	−0.006 (4)	0.000	0.000
S3	0.0154 (15)	0.0154 (15)	0.015 (2)	0.002 (2)	−0.003 (2)	−0.003 (2)

### Geometric parameters (Å, °)

**Table:**

Hg1—S1	2.488 (4)	Cs1—S2^iv^	3.566 (4)
Hg1—S1^i^	2.540 (5)	Cs1—S3^v^	3.609 (7)
Hg1—S1^ii^	2.549 (5)	Cs1—S3	3.627 (7)
Hg1—S3^iii^	2.7754 (15)	Cs1—Hg1^viii^	4.1659 (17)
Hg1—Cs1^iv^	4.1659 (17)	Cs1—Hg1^ii^	4.1659 (17)
Hg1—Cs1^iii^	4.2013 (18)	Cs1—Cs1^x^	4.1895 (18)
Hg1—Cs1	4.2412 (12)	Cs1—Cs1^v^	4.1895 (18)
Hg2—S3	2.367 (7)	Cs1—Hg1^i^	4.2013 (18)
Hg2—S2	2.377 (5)	S1—Hg1^iii^	2.540 (5)
Hg2—S3^v^	3.004 (7)	S1—Hg1^iv^	2.549 (5)
Hg2—Cs1^i^	4.2392 (19)	S1—Cs1^iv^	3.526 (5)
Hg2—Cs1^vi^	4.2392 (18)	S1—Cs1^iii^	3.561 (5)
Hg2—Cs1	4.631 (2)	S2—Hg2^xi^	2.377 (5)
Hg2—Cs1^ii^	4.640 (2)	S2—Cs1^ii^	3.566 (4)
Hg2—Cs1^vii^	4.640 (2)	S2—Cs1^vii^	3.566 (4)
Cs1—S1^viii^	3.526 (5)	S3—Hg1^ix^	2.7754 (14)
Cs1—S1^ii^	3.526 (5)	S3—Hg1^i^	2.7754 (14)
Cs1—S1^ix^	3.561 (5)	S3—Hg2^x^	3.004 (7)
Cs1—S1^i^	3.561 (5)	S3—Cs1^x^	3.609 (7)
			
S1—Hg1—S1^i^	121.0 (2)	S1^ix^—Cs1—S2^iv^	77.06 (11)
S1—Hg1—S1^ii^	120.6 (2)	S1^i^—Cs1—S2^iv^	77.06 (11)
S1^i^—Hg1—S1^ii^	110.83 (15)	S1^viii^—Cs1—S3^v^	71.57 (10)
S1—Hg1—S3^iii^	104.78 (15)	S1^ii^—Cs1—S3^v^	71.57 (10)
S1^i^—Hg1—S3^iii^	96.43 (18)	S1^ix^—Cs1—S3^v^	111.39 (9)
S1^ii^—Hg1—S3^iii^	95.74 (17)	S1^i^—Cs1—S3^v^	111.39 (9)
S1—Hg1—Cs1^iv^	57.59 (10)	S2^iv^—Cs1—S3^v^	164.39 (19)
S1^i^—Hg1—Cs1^iv^	92.09 (11)	S1^viii^—Cs1—S3	111.21 (9)
S1^ii^—Hg1—Cs1^iv^	148.09 (10)	S1^ii^—Cs1—S3	111.21 (9)
S3^iii^—Hg1—Cs1^iv^	58.64 (13)	S1^ix^—Cs1—S3	70.96 (10)
S1—Hg1—Cs1^iii^	57.74 (11)	S1^i^—Cs1—S3	70.96 (10)
S1^i^—Hg1—Cs1^iii^	148.92 (10)	S2^iv^—Cs1—S3	124.85 (19)
S1^ii^—Hg1—Cs1^iii^	91.15 (11)	S3^v^—Cs1—S3	70.76 (11)
S3^iii^—Hg1—Cs1^iii^	58.46 (14)	Hg1^viii^—Cs1—Hg1^ii^	81.48 (4)
Cs1^iv^—Hg1—Cs1^iii^	60.09 (3)	S1^viii^—Cs1—Hg1^i^	146.72 (8)
S1—Hg1—Cs1	168.11 (10)	S1^ii^—Cs1—Hg1^i^	77.73 (8)
S1^i^—Hg1—Cs1	56.93 (10)	S1^ix^—Cs1—Hg1^i^	103.37 (9)
S1^ii^—Hg1—Cs1	56.13 (10)	S1^i^—Cs1—Hg1^i^	36.20 (6)
S3^iii^—Hg1—Cs1	87.09 (12)	S2^iv^—Cs1—Hg1^i^	111.51 (11)
Cs1^iv^—Hg1—Cs1	132.09 (4)	S3^v^—Cs1—Hg1^i^	80.02 (9)
Cs1^iii^—Hg1—Cs1	131.02 (4)	S3—Cs1—Hg1^i^	40.70 (2)
S3—Hg2—S2	168.5 (3)	Hg1^viii^—Cs1—Hg1^i^	110.24 (4)
S3—Hg2—S3^v^	101.9 (2)	Hg1^ii^—Cs1—Hg1^i^	60.09 (3)
S2—Hg2—S3^v^	89.7 (2)	Cs1^x^—Cs1—Hg1^i^	59.53 (3)
S3—Hg2—Cs1^i^	92.64 (11)	Cs1^v^—Cs1—Hg1^i^	120.47 (3)
S2—Hg2—Cs1^i^	80.40 (11)	Hg1—S1—Hg1^iii^	104.31 (17)
S3^v^—Hg2—Cs1^i^	125.85 (4)	Hg1—S1—Hg1^iv^	104.03 (17)
S3—Hg2—Cs1^vi^	92.64 (11)	Hg1^iii^—S1—Hg1^iv^	110.83 (15)
S2—Hg2—Cs1^vi^	80.40 (11)	Hg1—S1—Cs1^iv^	85.86 (12)
S3^v^—Hg2—Cs1^vi^	125.85 (4)	Hg1^iii^—S1—Cs1^iv^	155.98 (17)
Cs1^i^—Hg2—Cs1^vi^	104.84 (5)	Hg1^iv^—S1—Cs1^iv^	86.99 (14)
S3—Hg2—Cs1	50.68 (17)	Hg1—S1—Cs1^iii^	86.05 (12)
S2—Hg2—Cs1	140.87 (19)	Hg1^iii^—S1—Cs1^iii^	86.37 (14)
S3^v^—Hg2—Cs1	51.17 (12)	Hg1^iv^—S1—Cs1^iii^	156.55 (16)
Cs1^i^—Hg2—Cs1	119.97 (2)	Cs1^iv^—S1—Cs1^iii^	72.47 (8)
Cs1^vi^—Hg2—Cs1	119.97 (2)	Hg2—S2—Hg2^xi^	112.2 (4)
S3—Hg2—Cs1^ii^	133.26 (3)	Hg2—S2—Cs1^ii^	100.76 (3)
S2—Hg2—Cs1^ii^	49.02 (6)	Hg2^xi^—S2—Cs1^ii^	100.76 (3)
S3^v^—Hg2—Cs1^ii^	77.46 (8)	Hg2—S2—Cs1^vii^	100.76 (3)
Cs1^i^—Hg2—Cs1^ii^	56.09 (3)	Hg2^xi^—S2—Cs1^vii^	100.76 (3)
Cs1^vi^—Hg2—Cs1^ii^	126.15 (3)	Cs1^ii^—S2—Cs1^vii^	140.9 (3)
Cs1—Hg2—Cs1^ii^	111.95 (3)	Hg2—S3—Hg1^ix^	98.40 (15)
S3—Hg2—Cs1^vii^	133.26 (3)	Hg2—S3—Hg1^i^	98.40 (15)
S2—Hg2—Cs1^vii^	49.02 (6)	Hg1^ix^—S3—Hg1^i^	156.8 (2)
S3^v^—Hg2—Cs1^vii^	77.46 (8)	Hg2—S3—Hg2^x^	101.9 (2)
Cs1^i^—Hg2—Cs1^vii^	126.15 (3)	Hg1^ix^—S3—Hg2^x^	96.04 (16)
Cs1^vi^—Hg2—Cs1^vii^	56.09 (3)	Hg1^i^—S3—Hg2^x^	96.04 (16)
Cs1—Hg2—Cs1^vii^	111.95 (3)	Hg2—S3—Cs1^x^	169.7 (3)
Cs1^ii^—Hg2—Cs1^vii^	92.79 (5)	Hg1^ix^—S3—Cs1^x^	80.31 (13)
S1^viii^—Cs1—S1^ii^	108.22 (17)	Hg1^i^—S3—Cs1^x^	80.31 (13)
S1^viii^—Cs1—S1^ix^	72.47 (8)	Hg2^x^—S3—Cs1^x^	88.40 (19)
S1^ii^—Cs1—S1^ix^	176.94 (13)	Hg2—S3—Cs1	99.0 (2)
S1^viii^—Cs1—S1^i^	176.94 (13)	Hg1^ix^—S3—Cs1	80.83 (13)
S1^ii^—Cs1—S1^i^	72.47 (8)	Hg1^i^—S3—Cs1	80.83 (13)
S1^ix^—Cs1—S1^i^	106.68 (16)	Hg2^x^—S3—Cs1	159.2 (2)
S1^viii^—Cs1—S2^iv^	99.89 (11)	Cs1^x^—S3—Cs1	70.76 (11)
S1^ii^—Cs1—S2^iv^	99.89 (11)		

Symmetry codes: (i) *y*−1/2, −*x*+1/2, *z*+1/2; (ii) *y*−1/2, −*x*+1/2, *z*−1/2; (iii) −*y*+1/2, *x*+1/2, *z*−1/2; (iv) −*y*+1/2, *x*+1/2, *z*+1/2; (v) *x*, *y*, *z*−1; (vi) −*y*+1/2, *x*−1/2, *z*+1/2; (vii) −*y*+1/2, *x*−1/2, *z*−1/2; (viii) −*x*+1/2, *y*−1/2, *z*−1/2; (ix) −*x*+1/2, *y*−1/2, *z*+1/2; (x) *x*, *y*, *z*+1; (xi) −*x*, −*y*, *z*.
